# Risk factors for the development of idiopathic macular hole: a nationwide population-based cohort study

**DOI:** 10.1038/s41598-022-25791-1

**Published:** 2022-12-16

**Authors:** Sungsoon Hwang, Se Woong Kang, Sang Jin Kim, Jaehwan Choi, Ki Young Son, Dong Hui Lim, Dong Wook Shin, DooSeok Choi, Yoosoo Chang, Seungho Ryu, Juhee Cho

**Affiliations:** 1 Department of Ophthalmology, Samsung Medical Center, Sungkyunkwan University School of Medicine, Seoul, Republic of Korea; 2 Department of Clinical Research Design and Evaluation, Samsung Advanced Institute for Health Sciences and Technology, Sungkyunkwan University, Seoul, Republic of Korea; 3 Department of Family Medicine and Supportive Care Center, Samsung Medical Center, Sungkyunkwan University School of Medicine, Seoul, Republic of Korea; 4 Department of Obstetrics and Gynecology, Samsung Medical Center, Sungkyunkwan University School of Medicine, Seoul, Republic of Korea; 5 Center for Cohort Studies, Total Healthcare Center, Kangbuk Samsung Hospital, Sungkyunkwan University School of Medicine, Seoul, Republic of Korea; 6 Department of Occupational and Environmental Medicine, Kangbuk Samsung Hospital, Sungkyunkwan University School of Medicine, Seoul, Republic of Korea; 7 Center for Clinical Epidemiology, Samsung Medical Center, Seoul, Republic of Korea; 8 Departments of Epidemiology and Medicine, Welch Center for Prevention, Epidemiology, and Clinical Research, Johns Hopkins University Bloomberg School of Public Health, Baltimore, MD USA

**Keywords:** Risk factors, Retinal diseases, Vitreous detachment, Epidemiology

## Abstract

This nationwide population-based cohort study searched for demographic, comorbid, behavioral, and reproductive risk factors for idiopathic macular hole (MH) development using data provided by the Korean National Health Insurance Service. A total of 4,496,867 individuals aged 50–79 years who participated in the Korean National Health Screening Program in 2013 or 2014 were included. Participants were followed up until December 2018, and incident cases of idiopathic MH were identified. Prospective associations between incident idiopathic MH and various covariates were investigated using multivariable-adjusted Cox proportional hazard models. During an average follow-up period of 4.91 years, 3054 patients were newly diagnosed with idiopathic MHs. Women showed greater risk (hazard ratio of 1.71) and earlier presentation of idiopathic MH than men. Compared to the normal body mass index group, the obese group (≥ 25 kg/m^2^) showed a significantly lower risk of idiopathic MH. Among postmenopausal women, those with two or more children showed a greater risk of idiopathic MH than those who had not been pregnant, with a hazard ratio of 1.80. In conclusion, idiopathic MH occurred earlier and greater in women. Childbirth were associated with an increased risk of MH development, and obesity was associated with a lower risk of MH.

## Introduction

The macular hole (MH) is a full-thickness defect of the retinal tissue occurring at the center of the macula. MH can occur secondary to diabetic retinopathy, pathologic myopia, and other ocular condition, but most MH cases develop without obvious secondary cause and this condition is referred to as idiopathic MH. Idiopathic MH commonly affects people over 60 years of age and is caused by anteroposterior vitreomacular traction resulting from anomalous posterior vitreous detachment^1^. The prevalence of idiopathic MH ranges from 0.02 to 0.33%^2,3^, and the incidence rate of idiopathic MH in the general population was reported to be 7.80 per 100,000 person-years in the Olmsted County^4^ and 3.14 per 100,000 person-years in South Korea^5^. Approximately 10–16% of patients have MH in both eyes^6,7^, which implies that certain individuals have a greater tendency to develop MH. Nevertheless, the reason why some individuals have stronger anteroposterior vitreomacular traction and eventually progress to idiopathic MH is poorly understood.

Previous epidemiological studies attempted to identify the risk factors of MH and revealed that older age and female sex are significant risk factors for MH formation, with a reported female-to-male ratio from 2.0 to 3.3 from population-based studies^4,5^. However, risk factors for developing idiopathic MH other than age and sex are not yet known. Specifically, much remains unknown for the reasons why the disease develops frequently in postmenopausal women. A limited number of epidemiological studies have investigated other risk factors for MH development. Among those studies, the results were often inconsistent and limited by case–control design or the small number of study subjects, less than or about a hundred cases of MH^8,9^. Recently, a large-scale cohort study in the United States investigated the risk factors for developing idiopathic MH; however, did not sufficiently evaluate covariates such as behavioral or female reproductive factors^10^.

Herein, we performed a comprehensive risk factor analysis including demographic, comorbid, behavioral, and female reproductive factors from a nationwide population-based cohort to provide epidemiological basis for MH development and advance our understanding of the pathophysiology.

## Methods

### Setting

This was a nationwide, population-based, retrospective cohort study that used authorized data from the Korean National Health Insurance Service (NHIS) database. The study adhered to the tenets of the Declaration of Helsinki and was approved by the Institutional Review Board of Samsung Medical Center, Seoul, Republic of Korea (IRB File Number 2020-11-074). The board waived the requirement for informed consent based on the use of de-identified public data and the retrospective design of the study.

The NHIS is the insurer in South Korea that provides mandatory universal medical care and holds all medical information and health claims data in the country. The NHIS runs the National Health Screening Program (NHSP)^11^, a free biennial general health examination provided to all Koreans aged above 40 years. The NHIS also extends the National Cancer Screening Program (NCSP) for those receiving general health examinations through the NHSP^12^. The NCSP provides examinations for the stomach, liver, colorectal, breast, and cervical cancer for all registered individuals at the age indicated for cancer screening (breast cancer examination beginning at 40 years of age).

The NHIS database stores data on demographic information (age, sex, income level, date of death); health claims (date of clinical visit, diagnosis code defined by the Korean Classification of Diseases seventh revision [KCD-7], prescription records); and those from the NHSP and NCSP (participants’ responses to questionnaires, anthropometric measurements, laboratory test results) of all registered Koreans. This database has been widely used in previous studies that identified risk factors for various diseases^13,14^. Detailed information regarding the database profiles has been provided elsewhere^11,12^.

### Study design and participants

**Figure 1:**
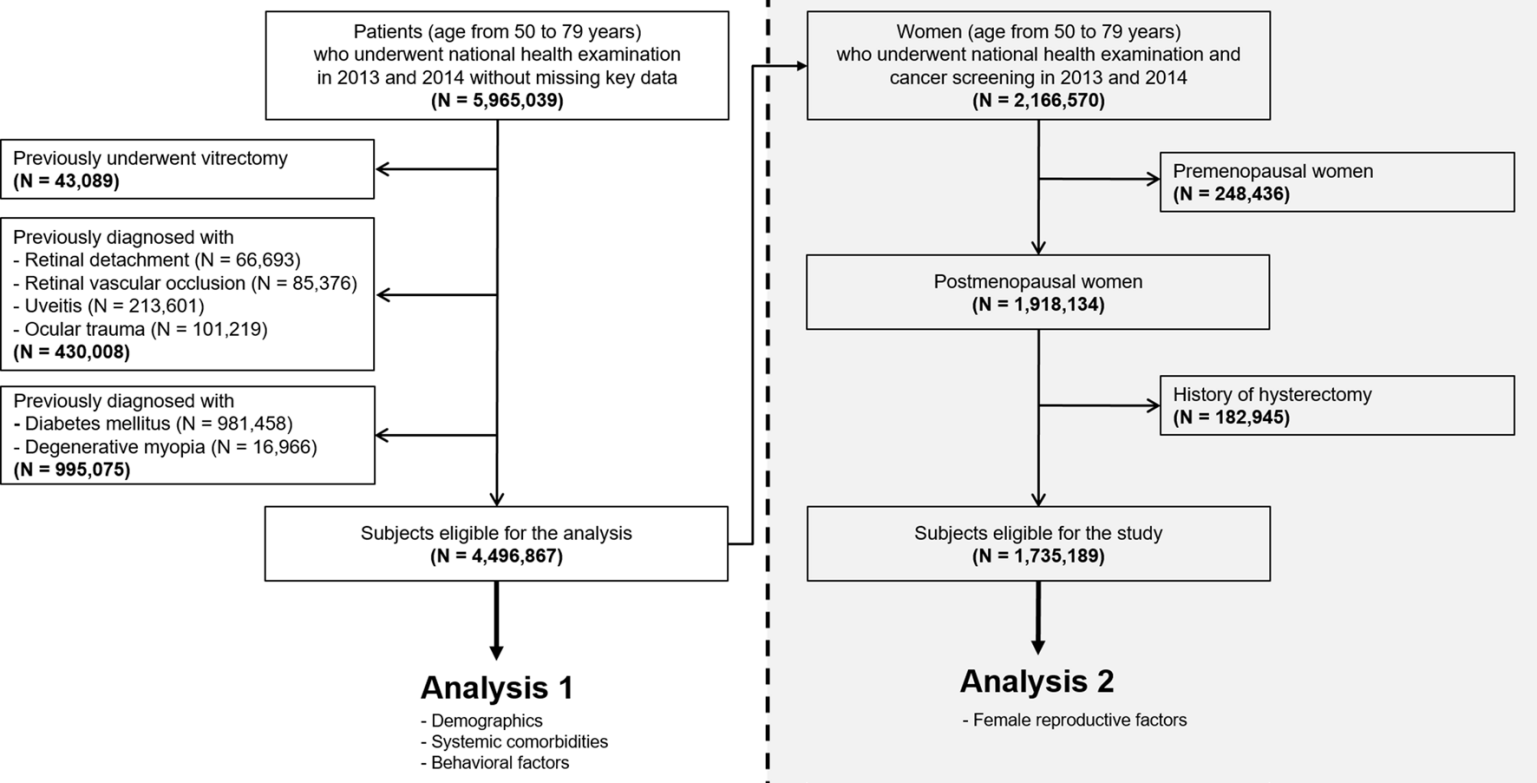
Flow chart of the cohort study design.

This study included two separate analyses (Fig. 1). In Analysis 1, we included both male and female participants who underwent NHSP, and investigated demographic, comorbid, and behavioral risk factors for MH development. In Analysis 2, only postmenopausal women who underwent NCSP were included to identify the association between female reproductive factors and the risk of MH development.

Among the entire South Korean population, participants who underwent national health examinations in 2013 and 2014 were identified. We included participants aged 50–79 years at baseline as most cases of posterior vitreous detachment would have already occurred in those over 80 years, and MH occurring before 50 years would have a secondary cause for MH development. Therefore, a total of 5,965,039 participants aged 50–79 years who participated in NHSP in 2013 and 2014 were identified. Individuals who had a history of vitrectomy (surgical code: S5121, S5122) from 2002 to the date of the examination were excluded from the study (n = 43,089) as they would not have vitreous that caused vitreofoveal traction and MH development. Individuals who had been diagnosed with retinal detachment (diagnostic code: H33), retinal vascular occlusion (diagnostic code: H34), uveitis (diagnostic code: H20, H30), and ocular trauma (diagnostic code: S05) from 2002 to the date of the examination were also excluded (n = 430,008) as they could cause secondary MH development. Individuals with diabetes mellitus at baseline and previous history of degenerative myopia (diagnostic code: H44.2) were then excluded as they are related to development of diabetic and myopic MH. Finally, 4,496,867 participants were included in Analysis 1.

Among those included in Analysis 1, 2,166,570 women underwent NCSP with NHSP and 1,918,134 postmenopausal women were identified. Individuals who had a history of hysterectomy (n = 182,945) were excluded from the study as they could have undergone simultaneous oophorectomy, influencing the participant's exposure to female hormones. Finally, 1,735,189 postmenopausal women were included in Analysis 2.

### Clinical data and reproductive factors

Comorbid hypertension, diabetes, and dyslipidemia were identified based on the followings; (1) self-reported questionnaire responses; (2) health screening measurement results of blood pressure (hypertension, systolic blood pressure ≥ 140 mmHg or diastolic blood pressure ≥ 90 mmHg), fasting glucose (diabetes, fasting blood glucose levels ≥ 126 mg/dL), and total cholesterol (dyslipidemia, ≥ 240 mg/dL); (3) the presence of diagnostic codes (KCD-7 code: I15 for hypertension, E11–E14 for diabetes, E78 for dyslipidemia) combined with medication prescription codes within a year before the health screening examination. The history of stroke and heart disease was identified using self-reporting based on the NHSP questionnaire. The presence of chronic kidney disease was defined as a glomerular filtration rate of < 60 mL/min/1.73 m^2^ measured using blood sampling. Income level was categorized into quartiles according to the insurance premium level, which was determined by household income.

Data on health-related behaviors were collected using the NHSP questionnaire. Smoking status was divided into never-smokers and past/current smokers. Drinking habits were categorized into three levels: none, mild (< 6 times a week), and heavy (≥ 6 times a week). Those who had a moderate level of physical activity for more than 30 min a day for more than 5 days per week were categorized into the regular exercise group. The body mass index (BMI) was calculated as the weight (kg) divided by height squared (m^2^), and categorized as underweight (BMI < 18.5 kg/m^2^), normal weight (18.5 ≤ BMI < 23 kg/m^2^), overweight (23 ≤ BMI < 25 kg/m^2^), obese I (25 ≤ BMI < 30 kg/m^2^), and obese II (≥ 30 kg/m^2^), according to the Korean Society for the Study of Obesity^15^.

Reproductive factor variables were retrieved from the NCSP questionnaire responses. The questionnaire items included age at menarche and menopause, parity, history of hormone replacement therapy (HRT), and oral contraceptive pill use. Data on female reproductive factors were categorized as follows: age at menarche (< 14 years, 14–15 years, 16–17 years, and ≥ 18 years), age at menopause (< 40 years, 40–44 years, 45–49 years, 50–54 years, and ≥ 55 years), parity (0, 1, ≥ 2 children), duration of HRT (never, < 2 years, 2 to < 5 years, ≥ 5 years, and unknown), and duration of oral contraceptive pill use (never, < 1 year, ≥ 1 year, unknown).

### Identification of incident MH development and follow-up

Incident MH cases were defined as those with both the diagnostic code for MH (H35.33) and the surgical codes for vitrectomy (S5121 or S5122) in the same claim after the date of the baseline examination^5^. Individuals with prior history of MH would not have been included in the study population, as we excluded those who had vitrectomy for any vitreoretinal disorder before the baseline examination. The incident date was defined as the date of the earliest claim that corresponded to the criteria for each patient. Incident MH cases that occurred after the diagnosis of retinal detachment, retinal vascular occlusion, uveitis, and ocular trauma were not counted as they could be secondary MH cases. Patients were followed from the date of health check-up to the date of incident MH, death, or the end of the study period (December 31, 2018), whichever came first.

### Statistical analyses

The incidence rates of MH were calculated by dividing the number of incident cases by the total number of person-years. Hazard ratios (HRs) and 95% confidence intervals (CIs) were calculated using Cox proportional hazard models. The variables adjusted for each Cox model in Analysis 1 were as follows: model 1, age and sex; model 2, demographics (age, sex, income level) and comorbidities (hypertension, dyslipidemia, stroke, heart disease, and chronic kidney disease); model 3, demographics; comorbidities; and behavioral factors (smoking status, drinking habit, regular exercise, and BMI). In Analysis 2, the variables adjusted for each Cox model were as follows: model 1, age group; model 2, demographics (age, income level); comorbidities; and behavioral factors; model 3, demographics; comorbidities; behavioral factors; and reproductive factors (age at menarche, age at menopause, parity, duration of HRT, and duration of oral contraceptive pill). Additional sensitivity analyses were conducted in a broader population in which previous diabetes mellitus or degenerative myopia were not excluded. The purpose of sensitivity analyses was to examine the robustness of study results and to additionally evaluate the influence of diabetes mellitus and degenerative myopia on the development of overall MH (including diabetic MH and myopic MH). All statistical analyses were performed using SAS version 9.4 (SAS Institute Inc., Cary, NC, USA).

## Results

### Analysis 1: baseline characteristics

Table 1 presents the detailed baseline characteristics of the individuals studied in Analysis 1. The mean age of the participants was 59.4 years, and 54.1% were women. The average follow-up period was 4.92 years, and 2353 patients were newly diagnosed with idiopathic MH and underwent vitrectomy during the follow-up period.

**Table 1:** Baseline characteristics of the study subjects for Analysis 1.

Variables	Total (N = 4,496,867)	Macular hole development	
		No (N = 4,494,514)	Yes (N = 2353)
***1. Demographic factors***			
Age, years, mean ± SD^‡^	59.41 ± 7.41	59.40 ± 7.41	62.90 ± 6.45
**Age group, no. (%)**^**‡**^			
50–54 years	1,528,770 (34.00)	1,528,505 (34.01)	265 (11.26)
55–59 years	1,041,030 (23.15)	1,040,587 (23.15)	443 (18.83)
60–64 years	856,148 (19.04)	855,392 (19.03)	756 (32.13)
65–69 years	464,548 (10.33)	464,095 (10.33)	453 (19.25)
70–74 years	436,434 (9.71)	436,095 (9.70)	339 (14.41)
75–79 years	169,937 (3.78)	169,840 (3.78)	97 (4.12)
**Sex, no. (%)**^**‡**^			
Male	2,065,240 (45.93)	2,064,494 (45.93)	746 (31.70)
Female	2,431,627 (54.07)	2,430,020 (54.07)	1607 (68.30)
**Income, no. (%)**^**‡**^			
Q1 (lowest)	938,554 (20.87)	938,071 (20.87)	483 (20.53)
Q2	820,656 (18.25)	820,281 (18.25)	375 (15.94)
Q3	1,042,339 (23.18)	1,041,804 (23.18)	535 (22.74)
Q4 (highest)	1,695,318 (37.70)	1,694,358 (37.70)	960 (40.8)
***2. Systemic comorbidities***			
**Hypertension, no. (%)**^**‡**^			
No	2,955,357 (65.72)	2,953,909 (65.72)	1448 (61.54)
Yes	1,541,510 (34.28)	1,540,605 (34.28)	905 (38.46)
**Dyslipidemia, no. (%)**^**‡**^			
No	3,141,123 (69.85)	3,139,610 (69.85)	1513 (64.30)
Yes	1,355,744 (30.15)	1,354,904 (30.15)	840 (35.70)
**Stroke, no. (%)**			
No	4,439,536 (98.73)	4,437,210 (98.73)	2326 (98.85)
Yes	57,331 (1.27)	57,304 (1.27)	27 (1.15)
**Heart diseases, no. (%)**^**†**^			
No	4,360,107 (96.96)	4,357,848 (96.96)	2259 (96.01)
Yes	136,760 (3.04)	136,666 (3.04)	94 (3.99)
**Chronic kidney disease, no. (%)**^**‡**^			
No	4,273,275 (95.03)	4,271,079 (95.03)	2196 (93.33)
Yes	223,592 (4.97)	223,435 (4.97)	157 (6.67)
***3. Behavioral factors***			
**Smoking history, no. (%)**^**‡**^			
Never smoked	3,004,725 (66.82)	3,002,882 (66.81)	1843 (78.33)
Past smoker	787,301 (17.51)	786,964 (17.51)	278 (11.81)
Current smoker	704,841 (15.67)	704,668 (15.68)	232 (9.86)
**Drinking habit, no. (%)**^**‡**^			
None	2,845,669 (63.28)	2,843,924 (63.28)	1745 (74.16)
Mild	1,450,016 (32.25)	1,449,492 (32.25)	524 (22.27)
Heavy	201,182 (4.47)	201,098 (4.47)	84 (3.57)
Regular physical activity, no. (%)*			
No	3,429,801 (76.27)	3,428,019 (76.27)	1782 (75.73)
Yes	1,067,066 (23.73)	1,066,495 (23.73)	571 (24.27)
**Body mass index, no (%)**			
< 18.5 kg/m^2^	102,141 (2.27)	102,092 (2.27)	49 (2.08)
18.5 to < 23 kg/m^2^	1,641,839 (36.51)	1,640,933 (36.51)	906 (38.50)
23 to < 25 kg/m^2^	1,230,170 (27.36)	1,229,516 (27.36)	654 (27.79)
25 to < 30 kg/m^2^	1,390,748 (30.93)	1,390,070 (30.93)	678 (28.81)
≥ 30 kg/m^2^	131,969 (2.93)	131,903 (2.93)	66 (2.8)

*SD* standard deviation, *Q* quartile.

*p-value < 0.05; ^†^p-value < 0.01; ^‡^p-value < 0.001.

### Analysis 1: demographics, comorbidities, behavioral factors, and the risk of MH

Table 2 shows the incidence rates and HRs with 95% CIs for MH development according to various covariates. The incidence rate of idiopathic MH was 10.63 per 100,000 person-years. The age group from 65 to 69 years showed the highest risk of MH development during the follow-up period. In multivariable-adjusted analysis (model 3), female patients had a greater risk of idiopathic MH development with HR (95% CI) of 1.70 (1.50–1.93). Obese individuals (BMI ≥ 25 kg/m^2^) presented a lower risk of MH development than those with normal BMI (HR, 0.83; BMI, 25–30 kg/m^2^ and 0.80; BMI ≥ 30 kg/m^2^). Individuals in the highest income quartile had a greater risk of MH than those in the lowest income quartile with HR (95% CI) of 1.17 (1.05–1.30).

**Table 2:** Hazard ratios and 95% confidence intervals of demographic, comorbid, and behavioral factors for development of idiopathic macular hole.

	Subject no.	Case no.	Duration (person-years)	IR per 100,000 person-years	Model 1	Model 2	Model 3
					HR (95% CI)	HR (95% CI)	HR (95% CI)
***Overall***	4,496,867	2,353	22,131,331	10.63			
***1. Demographic factors***							
**Age**							
50–54 years	1,528,770	265	7,548,458	3.51	1.00 (ref)	1.00 (ref)	1.00 (ref)
55–59 years	1,041,030	443	5,146,479	8.61	2.47 (2.12–2.87)	2.48 (2.13–2.89)	2.47 (2.12–2.87)
60–64 years	856,148	756	4,193,970	18.03	5.08 (4.41–5.84)	5.14 (4.46–5.92)	5.07 (4.40–5.85)
65–69 years	464,548	453	2,287,607	19.80	5.65 (4.86–6.58)	5.70 (4.89–6.65)	5.58 (4.78–6.52)
70–74 years	436,434	339	2,137,607	15.86	4.50 (3.83–5.28)	4.50 (3.81–5.30)	4.37 (3.70–5.16)
75–79 years	169,937	97	817,210	11.87	3.37 (2.67–4.25)	3.37 (2.66–4.27)	3.24 (2.55–4.11)
**Sex**							
Male	2,065,240	746	10,150,307	7.35	1.00 (ref)	1.00 (ref)	1.00 (ref)
Female	2,431,627	1,607	11,981,024	13.41	1.80 (1.65–1.96)	1.78 (1.63–1.95)	1.70 (1.50–1.93)
**Income**							
Q1 (lowest)	938,554	483	4,637,207	10.42	1.00 (ref)	1.00 (ref)	1.00 (ref)
Q2	820,656	375	4,056,398	9.24	0.98 (0.86–1.12)	0.98 (0.86–1.12)	0.98 (0.86–1.12)
Q3	1,042,339	535	5,140,685	10.41	1.01 (0.90–1.15)	1.01 (0.90–1.15)	1.01 (0.89–1.14)
Q4 (highest)	1,695,318	960	8,297,042	11.57	1.19 (1.06–1.32)	1.18 (1.06–1.32)	1.17 (1.05–1.30)
***2. Systemic comorbidities***							
**Hypertension**							
No	2,955,357	1,448	14,555,862	9.95	1.00 (ref)	1.00 (ref)	1.00 (ref)
Yes	1,541,510	905	7,575,469	11.95	0.94 (0.86–1.02)	0.93 (0.85–1.01)	0.95 (0.87–1.04)
**Dyslipidemia**							
No	3,141,123	1,513	15,464,274	9.78	1.00 (ref)	1.00 (ref)	1.00 (ref)
Yes	1,355,744	840	6,667,057	12.60	1.05 (0.97–1.15)	1.07 (0.98–1.17)	1.08 (0.99–1.18)
**Stroke**							
No	4,439,536	2,326	21,854,521	10.64	1.00 (ref)	1.00 (ref)	1.00 (ref)
Yes	57,331	27	276,810	9.75	0.74 (0.50–1.08)	0.74 (0.51–1.08)	0.73 (0.50–1.07)
**Heart disease**							
No	4,360,107	2,259	21,466,614	10.52	1.00 (ref)	1.00 (ref)	1.00 (ref)
Yes	136,760	94	664,717	14.14	1.06 (0.86–1.30)	1.07 (0.86–1.32)	1.06 (0.86–1.31)
**Chronic kidney disease**							
No	4,273,275	2,196	21,036,488	10.44	1.00 (ref)	1.00 (ref)	1.00 (ref)
Yes	223,592	157	1,094,844	14.34	0.99 (0.84–1.16)	0.99 (0.84–1.17)	0.99 (0.84–1.17)
***3. Behavioral factors***							
**Smoking history**							
Never smoked	3,004,725	1,843	14,799,836	12.45	1.00 (ref)	1.00 (ref)	1.00 (ref)
Past smoker	787,301	287	3,873,776	7.41	0.99 (0.90–1.10)	0.99 (0.90–1.10)	0.98 (0.89–1.09)
Current smoker	704,841	232	3,457,720	6.71	0.92 (0.83–1.05)	0.92 (0.83–1.05)	0.92 (0.82–1.04)
**Drinking habit**							
None	2,845,669	1,745	13,993,501	12.47	1.00 (ref)	1.00 (ref)	1.00 (ref)
Mild	1,450,016	524	7,152,259	7.33	0.94 (0.85–1.05)	0.95 (0.85–1.06)	0.97 (0.86–1.08)
Heavy	201,182	84	985,572	8.52	1.00 (0.80–1.26)	1.02 (0.81–1.28)	1.07 (0.85–1.34)
**Regular physical activity**							
No	3,429,801	1,782	16,870,533	10.56	1.00 (ref)	1.00 (ref)	1.00 (ref)
Yes	1,067,066	571	5,260,798	10.85	1.04 (0.94–1.14)	1.03 (0.93–1.13)	1.01 (0.92–1.11)
**Body mass index**							
< 18.5 kg/m^2^	102,141	49	489,996	10.00	0.86 (0.64–1.14)	0.87 (0.65–1.16)	0.88 (0.66–1.18)
18.5 to < 23 kg/m^2^	1,641,839	906	8,072,792	11.22	1.00 (ref)	1.00 (ref)	1.00 (ref)
23 to < 25 kg/m^2^	1,230,170	654	6,068,647	10.78	0.94 (0.85–1.04)	0.93 (0.84–1.03)	0.93 (0.84–1.02)
25 to < 30 kg/m^2^	1,390,748	678	6,852,873	9.89	0.84 (0.76–0.92)	0.84 (0.76–0.93)	0.83 (0.75–0.92)
≥ 30 kg/m^2^	131,969	66	647,023	10.20	0.80 (0.66–0.98)	0.81 (0.67–0.99)	0.80 (0.66–0.98)

*IR* incidence rate, *HR* hazard ratio, *CI* confidence interval, *Q* quartile.

Model 1: adjusted for age and sex.

Model 2: adjusted for demographic factors (age, sex, income level) and systemic comorbidities (hypertension, dyslipidemia, stroke, heart disease, chronic kidney disease).

Model 3: adjusted for demographic factors, systemic comorbidities, and behavioral factors (smoking history, drinking habit, physical activity, body mass index).

Table 3 presents the HRs and 95% CIs of idiopathic MH according to sex and its interaction with covariates in the multivariable-adjusted model (model 3). Men showed the greatest risk of MH development in the age group of 65–69 years, while women had the highest risk in the age group of 60–64 years (p-value for interaction < 0.001). No other variables showed any significant interactions between men and women.

**Table 3:** Hazard ratios and 95% confidence intervals for risk factors associated with idiopathic macular hole development according to sex and its interaction with other covariates.

	HR (95% CI) in Model 3		p-value for interaction
	Male	Female	
***1. Demographic factors***			
**Age group**			< 0.001
50–54 years	1.00 (ref)	1.00 (ref)	
55–59 years	2.60 (1.87–3.6)	2.45 (2.06–2.92)	
60–64 years	6.93 (5.13–9.35)	4.60 (3.91–5.41)	
65–69 years	10.32 (7.59–14.03)	4.37 (3.63–5.27)	
70–74 years	9.75 (7.12–13.35)	2.97 (2.41–3.66)	
75–79 years	8.43 (5.72–12.42)	1.88 (1.34–2.62)	
**Income**			0.43
Q1 (lowest)	1.00 (ref)	1.00 (ref)	
Q2	1.12 (0.88–1.45)	0.95 (0.81–1.11)	
Q3	1.10 (0.87–1.39)	1.01 (0.87–1.17)	
Q4 (highest)	1.40 (1.13–1.73)	1.15 (1.01–1.31)	
***2. Systemic comorbidities***			
**Hypertension**			0.21
No	1.00 (ref)	1.00 (ref)	
Yes	1.04 (0.89–1.22)	0.92 (0.83–1.03)	
**Dyslipidemia**			0.32
No	1.00 (ref)	1.00 (ref)	
Yes	1.17 (0.99–1.38)	1.05 (0.95–1.17)	
**Stroke**			0.89
No	1.00 (ref)	1.00 (ref)	
Yes	0.74 (0.43–1.29)	0.70 (0.41–1.19)	
**Heart disease**			0.17
No	1.00 (ref)	1.00 (ref)	
Yes	0.87 (0.62–1.23)	1.18 (0.9–1.54)	
**Chronic kidney disease**			0.96
No	1.00 (ref)	1.00 (ref)	
Yes	1.00 (0.75–1.34)	1.01 (0.83–1.24)	
***3. Behavioral factors***			
**Smoking history**			0.45
Never smoked	1.00 (ref)	1.00 (ref)	
Past smoker	0.97 (0.88–1.08)	0.97 (0.59–1.60)	
Current smoker	1.02 (0.88–1.16)	0.76 (0.53–1.10)	
**Drinking habit**			0.56
None	1.00 (ref)	1.00 (ref)	
Mild	1.02 (0.88–1.20)	0.94 (0.81–1.10)	
Heavy	1.08 (0.84–1.39)	0.79 (0.41–1.52)	
**Regular physical activity**			0.37
No	1.00 (ref)	1.00 (ref)	
Yes	1.05 (0.90–1.24)	0.96 (0.86–1.08)	
**Body mass index**			0.77
< 18.5 kg/m^2^	1.01 (0.63–1.61)	0.81 (0.56–1.17)	
18.5 to < 23 kg/m^2^	1.00 (ref)	1.00 (ref)	
23 to < 25 kg/m^2^	0.92 (0.77–1.10)	0.95 (0.85–1.08)	
25 to < 30 kg/m^2^	0.91 (0.76–1.09)	0.83 (0.73–0.94)	
≥ 30 kg/m^2^	0.78 (0.47–1.35)	0.84 (0.66–1.06)	

*HR* hazard ratio, *CI* confidence interval, *Q* quartile.

Model 3: adjusted for demographic factors (age, sex, income level), systemic comorbidities (hypertension, dyslipidemia, stroke, heart disease, chronic kidney disease), and behavioral factors (smoking history, drinking habit, physical activity, body mass index).

### Analysis 2: baseline characteristics

Table 4 shows the detailed baseline characteristics of the postmenopausal women included in Analysis 2. The mean age of the participants was 60.9 years. The average follow-up period was 4.91 years, and 1567 patients were newly diagnosed with MH and underwent vitrectomy during the follow-up period. The average age at menarche and menopause were 16.1 and 50.7 years, respectively. Approximately 90% of the participants had two or more children, and 20% had previously received HRT.

**Table 4:** Baseline characteristics of the study subjects for Analysis 2.

Variables	Total (N = 1,735,189)	Macular hole development	
		No (N = 1,733,622)	Yes (N = 1567)
***1. Demographic factors***			
Age, years, mean ± SD^‡^	60.92 ± 7.25	60.92 ± 7.25	62.70 ± 5.88
**Age group, no. (%)**^**‡**^			
50–54 years	416,317 (23.99)	416,182 (24.01)	135 (8.62)
55–59 years	419,626 (24.18)	419,299 (24.19)	327 (20.87)
60–64 years	401,424 (23.13)	400,845 (23.12)	579 (36.95)
65–69 years	210,769 (12.15)	210,484 (12.14)	285 (18.19)
70–74 years	206,741 (11.91)	206,546 (11.91)	195 (12.44)
75–79 years	80,312 (4.63)	80,266 (4.63)	46 (2.94)
Income, no. (%)*			
Q1 (lowest)	399,640 (23.03)	399,284 (23.03)	356 (22.72)
Q2	314,892 (18.15)	314,653 (18.15)	239 (15.25)
Q3	411,337 (23.71)	410,956 (23.71)	381 (24.31)
Q4 (highest)	609,320 (35.12)	608,729 (35.11)	591 (37.72)
***2. Systemic comorbidities***			
**Hypertension, no. (%)**			
No	1,131,954 (65.24)	1,130,952 (65.24)	1002 (63.94)
Yes	603,235 (34.76)	602,670 (34.76)	565 (36.06)
**Dyslipidemia, no. (%)**			
No	1,098,111 (63.28)	1,097,140 (63.29)	971 (61.97)
Yes	637,078 (36.72)	636,482 (36.71)	596 (38.03)
**Stroke, no. (%)**			
No	1,714,441 (98.80)	1,712,892 (98.80)	1,549 (98.85)
Yes	20,748 (1.20)	20,730 (1.20)	18 (1.15)
Heart diseases, no. (%)*			
No	1,683,166 (97.00)	1,681,663 (97.00)	1,503 (95.92)
Yes	52,023 (3.00)	51,959 (3.00)	64 (4.08)
**Chronic kidney disease, no. (%)**			
No	1,628,826 (93.87)	1,627,373 (93.87)	1,453 (92.72)
Yes	106,363 (6.13)	106,249 (6.13)	114 (7.28)
***3. Behavioral factors***			
**Smoking history, no. (%)**^**†**^			
Never smoked	1,677,557 (96.68)	1,676,027 (96.68)	1,530 (97.64)
Past smoker	18,764 (1.08)	18,747 (1.08)	17 (1.08)
Current smoker	38,868 (2.24)	38,848 (2.24)	20 (1.28)
**Drinking habit, no. (%)**^**†**^			
None	1,484,984 (85.58)	1,483,604 (85.58)	1380 (88.07)
Mild	236,033 (13.60)	235,852 (13.60)	181 (11.55)
Heavy	14,172 (0.82)	14,166 (0.82)	6 (0.38)
**Regular physical activity, no. (%)**			
No	1,348,872 (77.74)	1,347,651 (77.74)	1221 (77.92)
Yes	386,317 (22.26)	385,971 (22.26)	346 (22.08)
**Body mass index, no (%)**			
< 18.5 kg/m^2^	40,543 (2.34)	40,509 (2.34)	34 (2.17)
18.5 to < 23 kg/m^2^	669,478 (38.58)	668,872 (38.58)	606 (38.67)
23 to < 25 kg/m^2^	454,396 (26.19)	453,969 (26.19)	427 (27.25)
25 to < 30 kg/m^2^	508,906 (29.33)	508,456 (29.33)	450 (28.72)
≥ 30 kg/m^2^	61,866 (3.57)	61,816 (3.57)	50 (3.19)
***4. Reproductive factors***			
Age at menarche, mean ± SD	16.12 ± 2.02	16.12 ± 2.02	16.23 ± 2.48
**Age at menarche in group, no. (%)**			
< 14 years	115,015 (6.63)	114,923 (6.63)	92 (5.87)
14–15 years	579,180 (33.38)	578,668 (33.38)	512 (32.67)
16–17 years	654,397 (37.71)	653,803 (37.71)	594 (37.91)
≥ 18 years	386,597 (22.28)	386,228 (22.28)	369 (23.55)
Age at menopause, mean ± SD*	50.70 ± 3.78	50.70 ± 3.78	50.92 ± 4.03
**Age at menopause in group, no. (%)**^**‡**^			
< 45 years	82,441 (4.75)	82,360 (4.75)	81 (5.170)
45–49 years	399,423 (23.02)	399,083 (23.02)	340 (21.70)
50–54 years	1,029,066 (59.31)	1,028,181 (59.31)	885 (56.48)
≥ 55 years	224,259 (12.92)	223,998 (12.92)	261 (16.66)
**Parity, no. (%)**^**‡**^			
Nulliparous	31,956 (1.84)	31,941 (1.84)	15 (0.96)
1 child	152,356 (8.78)	152,257 (8.78)	99 (6.32)
≥ 2 children	1,550,877 (89.38)	1,549,424 (89.37)	1,453 (92.72)
**Hormone replacement therapy, no. (%)**^**†**^			
Never used	1,379,231 (79.49)	1,378,017 (79.49)	1214 (77.47)
< 2 years	163,134 (9.40)	162,970 (9.40)	164 (10.47)
2 to < 5 years	69,820 (4.02)	69,757 (4.02)	63 (4.02)
≥ 5 years	55,306 (3.19)	55,232 (3.19)	74 (4.72)
Unknown	67,698 (3.90)	67,646 (3.90)	52 (3.32)
**Oral contraceptive pill use, no. (%)**			
Never used	1,399,964 (80.68)	1,398,704 (80.68)	1260 (80.41)
< 1 year	155,170 (8.94)	155,028 (8.94)	142 (9.06)
≥ 1 year	98,230 (5.66)	98,143 (5.66)	87 (5.55)
Unknown	81,825 (4.72)	81,747 (4.72)	78 (4.98)

*SD* standard deviation, *Q* quartile.

*p-value < 0.05; ^†^p-value < 0.01; ^‡^p-value < 0.001.

### Analysis 2: female reproductive factors and the risk of MH

Table 5 shows HRs and 95% CIs of MH development according to female reproductive factors. In the fully adjusted model (model 3), participants who had given birth once or more had a greater risk of MH development compared to those who were nulliparous, with HR (95% CIs) of 1.58 (0.98–2.56) in those with having one child and 1.91 (1.15–3.18) in those with two or more children. Age at menopause and the history of HRT were not associated with the future risk of MH.

**Table 5:** Hazard ratios and 95% confidence intervals for the association between reproductive factors and the risk of idiopathic macular hole development in postmenopausal women.

	Subject no.	Case no.	Duration (person-years)	IR per 100,000 person-years	Model 1	Model 2	Model 3
					HR (95% CI)	HR (95% CI)	HR (95% CI)
***Overall***	1,735,189	1,567	8,517,534	18.40			
***4. Reproductive factors***							
Age at menarche							
< 14 years	115,015	92	557,818	16.49	1.00 (ref)	1.00 (ref)	1.00 (ref)
14–15 years	579,180	512	2,824,545	18.13	1.00 (0.80–1.25)	1.00 (0.80–1.25)	1.00 (0.80–1.25)
16–17 years	654,397	594	3,221,455	18.44	0.91 (0.73–1.14)	0.92 (0.73–1.14)	0.91 (0.73–1.13)
≥ 18 years	386,597	369	1,913,716	19.28	0.90 (0.71–1.13)	0.90 (0.72–1.14)	0.90 (0.71–1.13)
**Age at menopause**							
< 45 years	82,441	81	408,474	19.83	1.00 (ref)	1.00 (ref)	1.00 (ref)
45–49 years	399,423	340	1,967,163	17.28	0.93 (0.73–1.19)	0.93 (0.73–1.18)	0.92 (0.72–1.18)
50–54 years	1,029,066	885	5,042,493	17.55	0.94 (0.75–1.18)	0.93 (0.74–1.18)	0.93 (0.74–1.17)
≥ 55 years	224,259	261	1,099,405	23.74	0.98 (0.76–1.26)	0.98 (0.76–1.26)	0.99 (0.77–1.27)
**Parity**							
Nulliparous	31,956	15	155,296	9.66	1.00 (ref)	1.00 (ref)	1.00 (ref)
1 child	152,356	99	741,364	13.35	1.56 (0.97–2.53)	1.57 (0.97–2.55)	1.58 (0.98–2.56)
≥ 2 children	1,550,877	1,453	7,620,874	19.07	1.88 (1.13–3.12)	1.88 (1.13–3.13)	1.91 (1.15–3.18)
**Hormone replacement therapy**							
Never used	1,379,231	1,214	6,767,754	17.94	1.00 (ref)	1.00 (ref)	1.00 (ref)
< 2 years	163,134	164	802,633	20.43	1.14 (0.97–1.35)	1.13 (0.96–1.33)	1.14 (0.97–1.35)
2 to < 5 years	69,820	63	343,927	18.32	0.97 (0.75–1.25)	0.96 (0.74–1.23)	0.97 (0.75–1.25)
≥ 5 years	55,306	74	273,149	27.09	1.25 (0.99–1.58)	1.23 (0.97–1.56)	1.25 (0.98–1.58)
Unknown	67,698	52	330,071	15.75	0.87 (0.66–1.15)	0.87 (0.66–1.15)	0.85 (0.64–1.15)
**Oral contraceptive pill use**							
Never used	1,399,964	1,260	6,868,997	18.34	1.00 (ref)	1.00 (ref)	1.00 (ref)
< 1 year	155,170	142	762,769	18.62	0.97 (0.81–1.15)	0.97 (0.82–1.16)	0.95 (0.80–1.14)
≥ 1 year	98,230	87	485,202	17.93	0.88 (0.71–1.10)	0.89 (0.72–1.11)	0.88 (0.71–1.09)
Unknown	81,825	78	400,567	19.47	1.02 (0.81–1.28)	1.02 (0.81–1.29)	1.08 (0.85–1.37)

*IR* incidence rate, *HR* hazard ratio, *CI* confidence interval, *Q* quartile.

Model 1: adjusted for age.

Model 2: adjusted for demographic factors (age, income level), systemic comorbidities (hypertension, dyslipidemia, stroke, heart disease, chronic kidney disease), and behavioral factors (smoking history, drinking habit, physical activity, body mass index).

Model 3: adjusted for demographic factors, systemic comorbidities, behavioral factors, and female reproductive factors (age at menarche, age at menopause, parity, hormone replacement therapy, oral contraceptive pill).

### Sensitivity analyses

A total of 5,491,942 individuals were included in the additional sensitivity analyses. An average follow-up duration was 4.91 years and 3054 cases of MH developed. The age group from 65 to 69 years showed the highest risk of MH and obesity was associated with reduced risk of MH development (Supplemental Table 1). The history of giving birth was associated with greater risk of incident MH in postmenopausal women (Supplemental Table 2). The overall results of sensitivity analyses were similar to that of the main analyses. Diabetes mellitus and degenerative myopia were associated with increased risk of MH development.

## Discussion

This nationwide population-based cohort study revealed that age, sex, obesity, and parity influence the risk of idiopathic MH formation. And the risks of idiopathic MH according to age were significantly different between men and women. There has been a paucity of epidemiologic studies investigating the risk factors of MH development. Some previous studies investigated various parameters including behavioral factors, reproductive factors, and biochemical data; however, were limited by a small number of MH cases and a case–control design^8,9^. A recent cohort study from the United States included a fairly large number of participants; however, only investigated demographic parameters and comorbidities as risk factors for MH^10^. The present study addressed the lapses of these previous studies by including the largest number of participants and assessing various covariates comprehensively and would provide solid epidemiological evidence on the risk of MH in the available literature.

The incidence of idiopathic MH according to individuals’ age showed an inverted U pattern, with peak MH incidence in the age group of 65 to 69 years. MH occurs as a complication of posterior vitreous detachment^16^, and the peak MH incidence may reflect peak posterior vitreous detachment in that age group. Older individuals over 70 years of age are more likely to have complete posterior vitreous detachment, leading to a lower risk of MH development after the age at peak incidence. In the subgroup analysis (Table 3), women showed an earlier presentation of MH with the highest MH incidence in 60–64 years, while men had the most significant MH incidence in 65–69 years. The earlier presentation of MH in women has also been reported in previous studies^5,17,18^, and is attributed to the earlier progression of posterior vitreous detachment in women than in men^19^.

Female sex was a significant risk factor for idiopathic MH development, with an HR of 1.70 compared to males. Although the female preponderance in MH has been widely reported in various epidemiological studies^5,10^, the exact underlying mechanism for this predisposition is unknown. The most widely accepted biological explanation for more frequent MH development in women is the sudden drop in estrogen levels at menopause^20^. Estrogen plays a role in collagen and hyaluronic acid synthesis^21^. The sudden drop of estrogen level at menopause may cause loss of vitreous collagen and glycosaminoglycan, which could lead to premature and anomalous posterior vitreous detachment and result in more significant and earlier development of MH in postmenopausal women^10,20^. Although this estrogen theory prevails in the related literature, not much epidemiological or biological research supporting the theory exists and some studies even contradict it. Only one case–control study reported that the MH group had a lower proportion of current estrogen users compared with control participants with marginal statistical significance (p = 0.04)^8^. In contrast, another case–control study did not show any association between MH and HRT^9^. The estrogen levels in the vitreous gel of eyes with MH were not different from or higher than that of eyes with other retinal diseases in previous studies^22,23^, which implies null or even detrimental effects of estrogen on MH.

In the present study, the history of postmenopausal HRT was not associated with the risk of idiopathic MH development, which does not support the estrogen theory. Other parameters related to lifetime estrogen exposure (age at menarche and age at menopause) were not associated with the risk of idiopathic MH. Instead, parity was significantly associated with the risk of developing idiopathic MH. The Eye Disease Case–Control Study Group has previously reported the same association^8^. According to their study, the odds ratio (95% CI) of having MH was 1.80 (1.02–3.10) for those who had parity once or more compared to those who had never been pregnant. The biological reasons for this association are unclear. However, there has been a hypothesis expecting the linkage between parity and MH^24^. Mehdizadeh et al. suggested that a higher incidence of MH development might be attributable to relaxin, a hormone produced during pregnancy^24^. Relaxin facilitates the birth process by widening the pubic bone and softening the cervix by modulating collagen metabolism, inhibiting collagen synthesis, and enhancing its breakdown by increasing matrix metalloproteinases^25,26^. They suspected that pregnancy-induced relaxin secretion may affect collagen and hyaluronic acid metabolism in the vitreous as well, and may induce premature vitreous syneresis and early anomalous posterior vitreous detachment^24^. Accordingly, MH would be more prevalent and present at a younger age in women than in men. Moreover, the effect size (HR) of the female sex was similar to the effect size of parity (1.70 for women compared with men, and 1.58 for those who had one child and 1.91 for those who had two or more children compared with those without children, model 3), indicating that nulliparous females have a risk of MH development similar to that of males. Collectively, our study results suggest that parity, rather than estrogen exposure, such as the history of HRT or age at menarche and menopause, is closely associated with the risk of idiopathic MH. The precise pathophysiology underlying the linkage between parity and the increased risk of MH development should be further elucidated.

Additionally, those who were obese (BMI ≥ 25 kg/m^2^) had a significantly lower risk of developing idiopathic MH. The association between obesity and MH has not been previously reported. One possible explanation for this association is the association between obesity and myopia. Researchers have previously reported that people with higher BMI tend to be less myopic^27–29^. It has been suggested that obese individuals tend to have a greater amount of retrobulbar fat, which then increases retrobulbar pressure and hinders axial elongation of the globe^28,29^. Hence, obese individuals in the present study might be less myopic, which would lead to a lower risk of MH. We were unable to confirm the association between BMI and myopia and MH in the present study owing to the lack of data regarding refraction and axial length. Future epidemiological studies to confirm the association between BMI, myopia, and MH may provide insights into the underlying mechanisms of the association between obesity and MH.

The highest income quartile level was associated with an increased risk of MH undergoing vitrectomy. This might be because people with greater income may receive proper treatment for visual deterioration, while those with less income may be more likely to ignore symptoms, not visit ophthalmology clinics, and not receive proper management such as vitrectomy for MH. We adjusted income levels in multivariable analysis, which would have decreased bias that can be attributed to those with a lower income level not going to the hospital.

Sensitivity analyses including patients with diabetes and degenerative myopia draw results similar to the main analyses, which might be attributable to the fact that most of MH cases are idiopathic. Degenerative myopia was associated with an increased risk of MH development with an HR of 5.60. MH cases in degenerative myopia would mostly be myopic MH, which is related to a more complex pathogenetic mechanism than idiopathic MH. Posterior scleral ectasia related to degenerative myopia increases the degree of anteroposterior and tangential traction force which can lead to MH formation^30^. In addition, progressive retinal layer thinning caused by axial elongation makes the foveal structure vulnerable to traction force and further promotes the MH formation^31,32^. Diabetes was also associated with an increased risk of MH development. MH development in diabetic retinopathy has been widely reported, particularly in association with fibrovascular proliferation and macular edema^33,34^. The vitreous in diabetic eyes undergo abnormal collagen cross-linking^35^, and the retinal hemorrhage facilitates posterior hyaloid thickening^34^, which may lead to stronger vitreoretinal adhesion. Additionally, diabetic macular edema with intraretinal exudation/cyst may render the retina more vulnerable to vitreofoveal traction and lead to MH^34^. These may also have resulted in the greater incidence of MH in patients with diabetes in the present study.

This study conducted multiple analyses, and could have an increased risk of type 1 statistical error. However, the p-values achieved from Cox regression analyses were less than 0.001 for age, sex, and their interaction; and there was a clear trend that the degree of BMI and childbirth correlate with the risk of idiopathic MH. These implies that our results were not derived from a mere random error.

The present study has some limitations that need to be addressed. First, the incident MH was identified using claims data. Therefore, we could not perform medical chart level or imaging-based verification of the disease. The study also might have missed those who were unable to access the healthcare system or who declined to get vitrectomy for MH. Therefore, the incidence of MH might have been underestimated. These are the inherent limitations of the claims data-based study. Second, the KCD-7 code for MH can also be used for lamellar hole, so we might have included some cases in which vitrectomy was implemented in lamellar holes. However, vitrectomy is not generally performed for lamellar holes in South Korea, so the majority of our cases would be full-thickness MH, and very few would be lamellar hole cases. Third, the female reproductive parameters were collected using a self-reported questionnaire. Therefore, there is a possibility of bias attributable to inaccurate recall. Forth, as aforementioned, our study did not have data regarding ocular measures and other ocular comorbidities. In addition, it is not possible to perform per-eye-based analyses with NHIS database. Therefore, we were unable to investigate and confirm the ocular risk factors (e.g. refraction, cataract surgery, intravitreal injection) for MH development. Further studies addressing these limitations are required.

In conclusion, the present nationwide population-based cohort study revealed that age, sex, BMI, and parity in women significantly influenced the risk of future idiopathic MH development. This study would promote a better understanding of the disease entity and could serve as evidence for future research regarding the underlying biological mechanism of idiopathic MH.

## Supplementary Information

Supplementary Tables.

## Data Availability

The datasets analyzed in the current study were provided by the Korean NHIS. The data are available at https://nhiss.nhis.or.kr with the permission of the NHIS.
