# Supplementary material for: Risk factors for the development of idiopathic macular hole: a nationwide population-based cohort study
Source: Sci Rep. 2022 Dec 16;12:21778. doi: 10.1038/s41598-022-25791-1 (PMC9758209; doi:10.1038/s41598-022-25791-1)
Supplement: Supplementary file 1 — Supplementary Tables. [file 41598_2022_25791_MOESM1_ESM.pdf]

Risk Factors for the Development of Idiopathic Macular Hole: a nationwide population-based cohort study

Sungsoon Hwang, MD[1,2]; Se Woong Kang, MD, PhD[1]; Sang Jin Kim, MD, PhD[1]; Kyung Jun Choi, MD[1]; Ki Young Son, MD[1]; Dong Hui Lim, MD, PhD[1,2]; Dong Wook Shin, MD, PhD[2,3]; DooSeok Choi, MD, PhD[4]; Yoosoo Chang, MD, PhD[2.5.6]; Seungho Ryu, MD, PhD[2.5.6]; Juhee Cho, PhD[2,7,8]

[1]Department of Ophthalmology, Samsung Medical Center, Sungkyunkwan University School of Medicine, Seoul, Republic of Korea

[2]Department of Clinical Research Design & Evaluation, Samsung Advanced Institute for Health Sciences and Technology, Sungkyunkwan University, Seoul, Republic of Korea

[3]Department of Family Medicine and Supportive Care Center, Samsung Medical Center, Sungkyunkwan University School of Medicine, Seoul, Republic of Korea

[4]Department of Obstetrics and Gynecology, Samsung Medical Center, Sungkyunkwan University School of Medicine, Seoul, Republic of Korea

[5]Center for Cohort Studies, Total Healthcare Center, Kangbuk Samsung Hospital, Sungkyunkwan University School of Medicine, Seoul, Republic of Korea

[6]Department of Occupational and Environmental Medicine, Kangbuk Samsung Hospital, Sungkyunkwan University School of Medicine, Seoul, Republic of Korea

[7]Center for Clinical Epidemiology, Samsung Medical Center, Seoul, Republic of Korea

[8]Departments of Epidemiology and Medicine, Welch Center for Prevention, Epidemiology, and Clinical Research, Johns Hopkins University Bloomberg School of Public Health, Baltimore, MD, USA

Correspondence

Se Woong Kang, MD, PhD

Department of Ophthalmology, Samsung Medical Center, Sungkyunkwan University School of Medicine, #81 Irwon-ro, Gangnam-gu, Seoul 06351, Republic of Korea

Tel: 82-2-3410-3548, Fax: 82-2-3410-0074, E-mail: kangsewoong@gmail.com

Supplemental Table 1. Hazard ratios and 95% confidence intervals of demographic, comorbid, and behavioral factors for development of macular hole in the population in which previous diabetes mellitus or degenerative myopia were not excluded.

	Subject No.	Case No.	Duration (person-years)	IR per 100,000 person-years	Model 1	Model 2	Model 3
					HR (95% CI)	HR (95% CI)	HR (95% CI)
Overall	5,491,942	3,054	26,966,351	11.33			
Demographic Factors							
Age							
50–54 years	1,730,188	309	8,535,534	3.62	1.00 (ref)	1.00 (ref)	1.00 (ref)
55–59 years	1,234,304	540	6,093,589	8.86	2.47 (2.14–2.84)	2.47 (2.15–2.84)	2.45 (2.13–2.82)
60–64 years	1,076,680	955	5,265,062	18.14	4.95 (4.35–5.63)	4.95 (4.35–5.63)	4.88 (4.28–5.56)
65–69 years	612,907	627	3,009,824	20.83	5.74 (5.01–6.58)	5.69 (4.95–6.54)	5.56 (4.83–6.39)
70–74 years	600,321	492	2,927,719	16.80	4.58 (3.98–5.28)	4.48 (3.87–5.19)	4.34 (3.74–5.04)
75–79 years	237,542	131	1,134,623	11.55	3.14 (2.56–3.85)	3.07 (2.49–3.78)	2.94 (2.38–3.62)
Sex							
Male	2,619,345	1,001	12,825,270	7.80	1.00 (ref)	1.00 (ref)	1.00 (ref)
Female	2,872,597	2,053	14,141,081	14.52	1.82 (1.69–1.96)	1.81 (1.67–1.95)	1.71 (1.53–1.91)
Income							
Q1 (lowest)	1,156,693	639	5,697,779	11.21	1.00 (ref)	1.00 (ref)	1.00 (ref)
Q2	993,684	467	4,898,796	9.53	0.94 (0.83–1.06)	0.94 (0.83–1.06)	0.94 (0.83–1.06)
Q3	1,277,266	713	6,283,387	11.35	1.03 (0.92–1.14)	1.03 (0.92–1.14)	1.02 (0.92–1.14)
Q4 (highest)	2,064,299	1,235	10,086,389	12.24	1.15 (1.05–1.27)	1.15 (1.05–1.27)	1.14 (1.03–1.25)
Systemic Comorbidities							
Hypertension							
No	3,304,310	1,695	16,257,321	10.43	1.00 (ref)	1.00 (ref)	1.00 (ref)
Yes	2,187,632	1,359	10,709,029	12.69	0.95 (0.88–1.02)	0.92 (0.85–0.99)	0.94 (0.87–1.02)
Diabetes mellitus							
No	4,510,484	2,405	22,198,236	10.83	1.00 (ref)	1.00 (ref)	1.00 (ref)
Yes	981,458	649	4,768,115	13.61	1.11 (1.02–1.21)	1.12 (1.02–1.22)	1.14 (1.04–1.24)
Dyslipidemia							
No	3,545,060	1,765	17,428,907	10.13	1.00 (ref)	1.00 (ref)	1.00 (ref)
Yes	1,946,882	1,289	9,537,444	13.52	1.09 (1.01–1.17)	1.09 (1.01–1.17)	1.10 (1.02–1.19)
Stroke							
No	5,405,425	3,012	26,551,256	11.34	1.00 (ref)	1.00 (ref)	1.00 (ref)
Yes	86,517	42	415,095	10.12	0.80 (0.63–1.02)	0.79 (0.62–1.02)	0.79 (0.62–1.01)
Heart disease							
No	5,283,361	2,906	25,957,584	11.20	1.00 (ref)	1.00 (ref)	1.00 (ref)
Yes	208,581	148	1,008,767	14.67	1.05 (0.89–1.24)	1.04 (0.88–1.23)	1.03 (0.87–1.22)

Chronic kidney disease							
No	5,162,111	2,794	25,365,537	11.01	1.00 (ref)	1.00 (ref)	1.00 (ref)
Yes	329,831	260	1,600,814	16.24	1.08 (0.95–1.23)	1.08 (0.95–1.23)	1.08 (0.95–1.23)
Behavioral Factors							
Smoking history							
Never smoked	3,596,034	2,358	17,685,195	13.33	1.00 (ref)	1.00 (ref)	1.00 (ref)
Past smoker	1,004,044	379	4,923,237	7.70	0.96 (0.88–1.06)	0.96 (0.88–1.06)	0.95 (0.87–1.05)
Current smoker	891,864	317	4,357,919	7.27	0.91 (0.84–1.02)	0.91 (0.84–1.02)	0.91 (0.83–1.01)
Drinking habit							
None	3,471,817	2,265	17,033,278	13.30	1.00 (ref)	1.00 (ref)	1.00 (ref)
Mild	1,762,463	678	8,675,593	7.82	0.96 (0.87–1.05)	0.96 (0.87–1.06)	0.98 (0.89–1.08)
Heavy	257,662	111	1,257,480	8.83	1.00 (0.82–1.22)	1.01 (0.83–1.24)	1.06 (0.87–1.30)
Regular physical activity							
No	4,185,638	2,315	20,537,299	11.27	1.00 (ref)	1.00 (ref)	1.00 (ref)
Yes	1,306,304	739	6,429,052	11.49	1.03 (0.95–1.12)	1.02 (0.94–1.11)	1.00 (0.92–1.09)
Body mass index							
< 18.5 kg/m^2	116,473	59	554,621	10.64	0.86 (0.66–1.11)	0.87 (0.67–1.13)	0.89 (0.68–1.15)
18.5 to < 23 kg/m^2	1,903,375	1,129	9,333,425	12.10	1.00 (ref)	1.00 (ref)	1.00 (ref)
23 to < 25 kg/m^2	1,490,201	853	7,336,927	11.63	0.94 (0.86–1.03)	0.93 (0.85–1.02)	0.92 (0.84–1.01)
25 to < 30 kg/m^2	1,787,469	905	8,790,599	10.30	0.81 (0.74–0.88)	0.80 (0.73–0.88)	0.79 (0.72–0.86)
≥ 30 kg/m^2	194,424	108	950,779	11.36	0.82 (0.70–0.97)	0.81 (0.69–0.96)	0.80 (0.69–0.95)
Factors related to secondary macular hole							
Diabetes mellitus							
No	4,510,484	2,405	22,198,236	10.83	1.00 (ref)	1.00 (ref)	1.00 (ref)
Yes	981,458	649	4,768,115	13.61	1.11 (1.02–1.21)	1.12 (1.02–1.22)	1.14 (1.04–1.24)
Degenerative myopia							
No	5,474,976	2,993	26,883,093	11.13	1.00 (ref)	1.00 (ref)	1.00 (ref)
Yes	16966	61	83,258	73.27	5.69 (4.42–7.33)	5.65 (4.38–7.28)	5.60 (4.34–7.21)

IR, incidence rate; HR, hazard ratio; CI, confidence interval; Q, quartile

Model 1: adjusted for age and sex.

Model 2: adjusted for demographic factors (age, sex, income level) and systemic comorbidities (hypertension, dyslipidemia, stroke, heart disease, chronic kidney disease).

Model 3: adjusted for demographic factors, systemic comorbidities, and behavioral factors (smoking history, drinking habit, physical activity, body mass index).

Supplemental Table 2. Hazard ratios and 95% confidence intervals for the association between reproductive factors and the risk of macular hole development in postmenopausal female population in which previous diabetes mellitus or degenerative myopia were not excluded.

	Subject No.	Case No.	Duration (person-years)	IR per 100,000 person-years	Model 1	Model 2	Model 3
					HR (95% CI)	HR (95% CI)	HR (95% CI)
Overall	2,075,860	2,024	10,183,570	19.88			
Reproductive Factors							
Age at menarche							
< 14 years	133,574	122	647,809	18.83	1.00 (ref)	1.00 (ref)	1.00 (ref)
14–15 years	678,381	636	3,307,264	19.23	0.93 (0.77–1.13)	0.93 (0.77–1.13)	0.93 (0.77–1.13)
16–17 years	788,653	784	3,878,978	20.21	0.88 (0.73–1.07)	0.89 (0.73–1.08)	0.88 (0.73–1.07)
≥ 18 years	475,252	482	2,349,520	20.51	0.85 (0.70–1.04)	0.86 (0.70–1.05)	0.85 (0.70–1.04)
Age at menopause							
< 45 years	104,297	112	515,979	21.71	1.00 (ref)	1.00 (ref)	1.00 (ref)
45–49 years	477,320	443	2,349,030	18.86	0.92 (0.75–1.14)	0.91 (0.74–1.13)	0.91 (0.74–1.12)
50–54 years	1,215,979	1,120	5,955,327	18.81	0.92 (0.76–1.12)	0.91 (0.75–1.11)	0.91 (0.75–1.11)
≥ 55 years	278,264	349	1,363,234	25.60	0.98 (0.79–1.22)	0.98 (0.79–1.22)	0.99 (0.80–1.22)
Parity							
Nulliparous	37,399	20	181,742	11.00	1.00 (ref)	1.00 (ref)	1.00 (ref)
1 child	174,721	133	849,762	15.65	1.60 (1.00–2.56)	1.62 (1.01–2.58)	1.62 (1.01–2.59)
≥ 2 children	1,863,740	1,871	9,152,066	20.44	1.77 (1.14–2.75)	1.79 (1.15–2.78)	1.80 (1.16–2.81)
Hormone replacement therapy							
Never used	1,657,933	1,580	8,130,567	19.43	1.00 (ref)	1.00 (ref)	1.00 (ref)
< 2 years	189,090	209	930,143	22.47	1.12 (0.97–1.28)	1.11 (0.96–1.27)	1.11 (0.96–1.27)
2 to < 5 years	80,479	79	396,474	19.93	0.98 (0.78–1.23)	0.96 (0.77–1.20)	0.97 (0.77–1.21)
≥ 5 years	65,113	91	321,287	28.32	1.22 (0.99–1.51)	1.20 (0.97–1.48)	1.21 (0.97–1.49)
Unknown	83,245	65	405,099	16.05	0.82 (0.64–1.05)	0.82 (0.64–1.05)	0.80 (0.62–1.04)
Oral contraceptive pill use							
Never used	1,665,839	1,615	8,169,327	19.77	1.00 (ref)	1.00 (ref)	1.00 (ref)
< 1 year	186,299	190	915,279	20.76	1.00 (0.86–1.17)	1.01 (0.87–1.17)	0.99 (0.85–1.15)
≥ 1 year	123,241	117	607,941	19.25	0.88 (0.73–1.06)	0.89 (0.74–1.08)	0.88 (0.73–1.06)
Unknown	100,481	102	491,023	20.77	1.01 (0.83–1.24)	1.02 (0.83–1.24)	1.09 (0.88–1.35)

IR, incidence rate; HR, hazard ratio; CI, confidence interval; Q, quartile

Model 1: adjusted for age.

Model 2: adjusted for demographic factors (age, income level), systemic comorbidities (hypertension, dyslipidemia, stroke, heart disease, chronic kidney disease), and behavioral factors (smoking history, drinking habit, physical activity, body mass index).

Model 3: adjusted for demographic factors, systemic comorbidities, behavioral factors, and female reproductive factors (age at menarche, age at menopause, parity, hormone replacement therapy, oral contraceptive pill).
